# A *Varroa destructor* protein atlas reveals molecular underpinnings of developmental transitions and sexual differentiation*[Fn FN2]

**DOI:** 10.1074/mcp.RA117.000104

**Published:** 2017-09-22

**Authors:** Alison McAfee, Queenie W. T. Chan, Jay Evans, Leonard J. Foster

**Affiliations:** From the ‡Department of Biochemistry & Molecular Biology, Michael Smith Laboratories, University of British Columbia, 2125 East Mall, Vancouver, British Columbia, Canada V6T 1Z4;; §Bee Research Laboratory, Beltsville Agricultural Research Center—East, U.S. Department of Agriculture, Beltsville, MD, USA 20705-0000

## Abstract

*Varroa destructor* is the most economically damaging honey bee pest, weakening colonies by simultaneously parasitizing bees and transmitting harmful viruses. Despite these impacts on honey bee health, surprisingly little is known about its fundamental molecular biology. Here, we present a *Varroa* protein atlas crossing all major developmental stages (egg, protonymph, deutonymph, and adult) for both male and female mites as a web-based interactive tool (http://foster.nce.ubc.ca/varroa/index.html). We used intensity-based label-free quantitation to find 1,433 differentially expressed proteins across developmental stages. Enzymes for processing carbohydrates and amino acids were among many of these differences as well as proteins involved in cuticle formation. Lipid transport involving vitellogenin was the most significantly enriched biological process in the foundress (reproductive female) and young mites. In addition, we found that 101 proteins were sexually regulated and functional enrichment analysis suggests that chromatin remodeling may be a key feature of sex determination. In a proteogenomic effort, we identified 519 protein-coding regions, 301 of which were supported by two or more peptides and 169 of which were differentially expressed. Overall, this work provides a first-of-its-kind interrogation of the patterns of protein expression that govern the *Varroa* life cycle and the tools we have developed will support further research on this threatening honey bee pest.

The *Varroa destructor* mite is the most devastating pest for Western honey bees (*Apis mellifera*) (1–3). This obligate parasite feeds on honey bee hemolymph (blood), simultaneously weakening its host, suppressing the innate immune system, and transmitting debilitating viruses (see Rosenkranz *et al.* (4) for a comprehensive review on *Varroa* biology). *Varroa*'s natural host is the Eastern honey bee (*A. cerana*), and millions of years of coevolution have led *A. cerana* to develop various tolerance mechanisms, thereby minimizing the mite's negative impact on these colonies (5–7). However, in the mid-1900s, the mite jumped hosts to *A. mellifera*—the bee species that is most commonly used for active crop pollination today—which is less effective at defending itself (4, 6). Managed *A. mellifera* colonies infested with *Varroa* have shorter lifespans than uninfested colonies unless they are actively treated with miticides (8, 9), causing serious negative economic impacts (10–12).

Despite being responsible for significant colony losses, very little is known about the molecular biology of the *Varroa* mite. Since the egg, protonymph, and deutonymph life stages (Fig. 1) only exist when the foundress mite (reproductive female) is actively reproducing within capped honey bee brood comb (4), they are seldom observed and are tedious to sample. Furthermore, male mites (even as adults) die soon after the adult honey bee emerges, so even though they are obviously important factors in mite reproduction, our knowledge of their basic molecular biology is extremely limited. Research on *Varroa* has focused on its role as a vector for viruses (13–18), their response to pheromone cues (19–21), attempts to control it via RNAi (22–24), and host shifts (25). At the time of writing, there have only been two previous *Varroa* proteomic investigations, one of which focused on viral proteins (15) and the other identifying fewer than 700 proteins within one developmental stage (26). Global protein expression changes associated with developmental transitions and sexual differentiation are yet unknown.

**Fig. 1.**
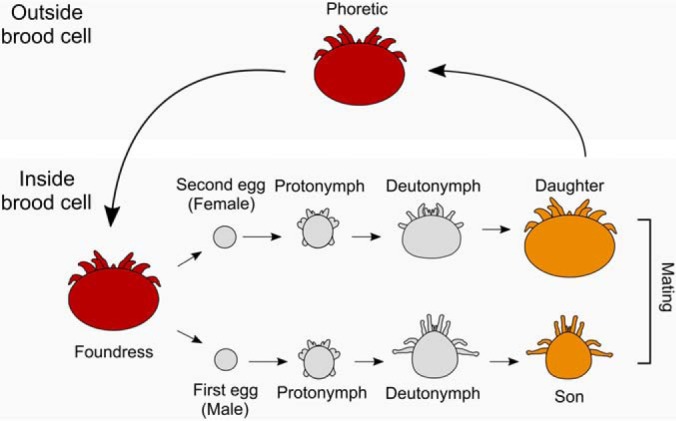
**Schematic representation of the mite life cycle.** All stages were included in this study (*n* = 3 for all) except the phoretic stage. For egg and protonymph stages, males and females are visually indistinguishable, so for these stages, sexes were pooled. Colors indicate melanization of the cuticle and sizes are proportional.

The *Varroa* genome was first sequenced in 2010 (1) and was accompanied by a provisional gene annotation that will be updated shortly. Gene annotations are living databases and, particularly with newly sequenced species, they undergo continuous refinement as more omic data become available. Unfortunately, the more evolutionarily distant a species is from well-annotated species typically used for orthology delineation and gene prediction training sets, the less accurate the predictions become. Such is the case for *Varroa.* Proteogenomics (27, 28) can help overcome this problem by sequencing the expressed protein regions in a relatively unbiased survey of the genomic landscape. Since protein expression is dynamic throughout an organism's life cycle, high-resolution omics data that cross developmental stages and sexes are very well-suited for this purpose.

Investigating global protein expression profiles throughout development of both sexes simultaneously provides a foundational understanding of *Varroa* biology and creates an opportunity to improve upon existing gene annotations. We present here the first *Varroa* proteome crossing all major developmental stages (egg, protonymph, deutonymph, adult) of both males and females, where distinguishable (Fig. 1). Through a proteogenomics effort, we identified 519 new protein-coding regions—301 of which are supported by two or more peptides. We also analyze the chemical properties of these sequences and their sequence similarity to other organisms to investigate reasons why underannotation continues to be a problem. We identified 3,102 proteins overall, nearly half (1,433) of which were significantly differentially expressed through development and 101 of which were differentially expressed between sexes. Functional enrichment suggested that carbohydrate and amino acid metabolism underpin developmental transitions, so we investigated proteins involved in glycolysis and the Krebs cycle in detail. Cuticle formation is clearly a process associated with mite aging, and closer analysis suggests the mites utilize different chitin structural proteins as they mature. In addition, chromatin remodeling and positive regulation of transcription may be key factors in sexual differentiation. Building on our previous honey bee protein atlas (29), we provide a web-based interactive platform (http://foster.nce.ubc.ca/varroa/index.html) where researchers can query proteins for visual displays of expression patterns, enabling further hypothesis generation and maximizing the utility of this information for the scientific community.

## EXPERIMENTAL PROCEDURES

### 

#### 

##### Sample Collection

*Varroa* mite families were collected from a single *A. mellifera* colony in the fall of 2016 in Vancouver, Canada. In a large-scale population genomics study, the authors found that the genetic variation of *Varroa* within colonies accounted for by far the largest fraction of genetic variation compared with between colonies and between apiaries (30); therefore, sampling mites from a single colony was sufficient. Eggs, foundresses, adult daughters, and adult sons were transferred directly to microfuge tubes using a soft paintbrush, whereas protonymphs and deutonymphs were transferred to a Petri dish and sorted under a dissecting microscope according to the identification guides available at http://idtools.org/id/mites/beemites and http://extension.msstate.edu/publications (publication number: P2826) *via* the University of Michigan and the Mississippi State University, respectively. Approximately 50 individuals were pooled for each replicate (seven developmental stages, *n* = 3 for each stage). All samples were immediately frozen at −72 °C until protein extraction.

##### Protein Preparation

Protein was extracted by homogenizing each mite stage with ceramic beads as previously described (31). Clarified lysate was precipitated overnight with four volumes of 100% ice cold acetone, and the pellet was washed twice with ice cold 80% acetone. After allowing residual acetone to evaporate (∼15 min), the protein pellet was solubilized in urea buffer (6 m urea, 2 m thiourea in 10 mm HEPES, pH 8) and ∼30 μg (determined via the Bradford Assay) was reduced, alkylated, and digested with Lys-C then trypsin as previously described (32). Peptides were acidified (one volume 1% TFA), desalted on a high capacity C18 STAGE tip (33), solubilized in Buffer A (0.1% formic acid), and quantified in technical triplicate using a peptide fluorometric assay (Pierce; cat: 23290).

##### Data Acquisition

2 μg of peptides per sample were analyzed on an EasynLC-1000 chromatography system (Thermo) coupled to a Bruker Impact II Q-TOF mass spectrometer. The LC C18 columns included a fritted trap column and pulled-tip, 50-cm analytical column produced and packed in-house (34, 35). Peptides were separated using a 165-min linear gradient of increasing Buffer B as specified in the LCParms.txt file embedded within the Bruker data folders (available at www.proteomexchange.org, accession: PXD006072). Buffers A and B were 0.1% formic acid and 0.1% formic acid, 80% acetonitrile, respectively. The instrument was set to the same parameters as described in our previous publication under “Analysis of PTMs” (34), except the scanned mass range was 200–2,000 *m/z*, the top 20 precursors were fragmented at a 5-Hz spectral rate, and the lower precursor intensity threshold was 300 counts.

#### Mass Spectrometry Data Analysis

##### Proteogenomics

For the proteogenomics analysis, the *Varroa* spectra were searched against a six-frame translation of the publicly available *Varroa* genome sequence (PRJNA33465) using MaxQuant (v.1.5.3.30) to identify new protein-coding regions (minimum ORF length was set to 100 amino acids). All viruses known to infect *A. mellifera* and *Varroa* were also included in the database. Honey bee proteins were not included after a follow-up sequence similarity analysis indicated that only five of the proteins identified in this search matched to bees. MaxQuant search settings included: trypsin cleavage specificity, two allowed missed cleavages, fixed carbamidomethyl modification, variable oxidated methionine and N-terminal acetylation, 0.07 Da precursor mass tolerance, 35 ppm fragment mass tolerance, and 1% protein and peptide FDR calculated based on reverse hits. The peptide (scores, modifications, precursor mass, and *m/z*) and protein (protein groups, accessions, number of assigned peptides, unique peptides, and % coverage) identification information contained within the main MaxQuant output files (summary.txt, peptides.txt, proteinGroups.txt, parameters.txt), and the protein database (165,951 entries) are available at PXD006072. We also include protein accessions, numbers of distinct peptides, and percentage protein coverage for each protein group in Supplemental Table 5. Annotated spectra are available through MS-viewer (search key: wuh30b9smr).

Peptides identified in the six-frame translation search but which were not present in the canonical protein database were used as anchors to retrieve the corresponding ORFs from the genome using a simple Perl script. This yielded 524 new protein-coding sequences. Of these, 301 were flanked by two or more peptides spanning at least 50 amino acid residues. We used a two-way ANOVA (factors: amino acid and new/known sequence origin) to compare amino acid composition between this set of 301 new protein-coding sequences and 902 sequences bounded by known peptides that were identified in the same six-frame translation search. We used these 902 sequences, which were also generated by the MaxQuant six-frame translation algorithm, because protein-coding sequences generated by more sophisticated algorithms (as with the canonical *Varroa* annotation) could generate different sequence properties simply due to the algorithm being different. These 902 sequences, however, were both a product of the six-frame translation and part of the canonical protein database. Next, we used the same approach to compare nucleotide positions within codons (factors: nucleotide position and sequence origin). We also compared the adenine and thymine frequency of the new coding regions, known coding regions, and genome sequences that were broken into 1-kb segments *in silico* (*n* = 384,129) using a one-way ANOVA (three levels) with a Tukey HSD (honestly significant difference) post-hoc test. We have included the Perl script modules used in these analyses as Supplemental File 1.

To survey these proteins for orthology with other species and to retrieve GO terms, we performed Blast2GO (v4.0) using default parameters. We reasoned that these sequences might have been missed in the *Varroa* annotation effort if they only share sequence similarity to evolutionarily distant species; therefore, we queried them against the nonredundant protein collection with no taxonomic restrictions. Five sequences showed significant homology to honey bee sequences and were removed from the list of new protein sequences, leaving 519 in total.

##### Protein Quantitation

We searched the mass spectrometry data using the same parameters as above, except label-free quantitation (LFQ) was enabled (with min ratio count = 1), and a composite protein database was used that included all proteins in the most recent *Varroa* gene annotation^1^ (the final protein database is included at the ProteomeXchange accession below), the 519 protein sequences identified above, all viral sequences known to infect *A. mellifera* or *Varroa*, and all proteins contained within the *A. mellifera* OGSv3.2 annotation. Since *A. mellifera* biological material is *Varroa's* sole food source, we expected to find a substantial number of honey bee proteins within our samples. The final database totaled 32,110 entries and is available at PXD006072, along with the MaxQuant peptide and protein identification information as described under “Proteogenomics.” We also include a table with protein accessions, numbers of distinct peptides, percentage protein coverage, and LFQ measurements (Supplemental Table 6). Honey bee proteins include an Amel tag in the accession, new protein-coding regions from the six-frame translation include a True or False tag in the accession (indicating the DNA template strand relative to the indicated contig), virus sequences are represented by a single gi number or Uniprot identifier, and all other sequences (excluding contaminants and reverse hits) belong to *Varroa*. Annotated spectra are available at MS-viewer (search key: msmx6z444s).

##### Experimental Design and Statistical Rationale

All seven developmental stages were collected in biological triplicate with ∼50 individuals pooled to create each replicate. Since each stage is a pool of many individuals, even a relatively low replication of *n* = 3 represents a large sample of the population. Only proteins with six or more observations (out of 21) were included in differential expression analysis across developmental stages. For the differential expression across sexes, this was relaxed to three or more observations to avoid excluding proteins that are stage and sex specific. Visual inspection of log-transformed LFQ intensity histograms confirmed the data for each replicate were distributed normally prior to analyzing with an ANOVA. For the developmental stage analysis, missing values were not imputed because they were too numerous, forcing a bimodal distribution upon imputation for some samples, which violates one assumption of the *t* test. For the sexual differentiation analysis, which excluded egg and protonymph stages, this was not the case, so missing values were imputed (width = 0.3, downshift = 1.5) to capture sex-specific proteins as previously described (36). Differential expression analysis across developmental stages was performed as previously described (31) except Perseus v1.5.6.0 was used, missing values were not imputed, and the ANOVA (one factor, seven levels) *p* values were Benjamini Hochberg-corrected at 5% FDR. For the analysis of sexually regulated genes, the female (deutonymph, adult daughter, and foundress) and male (deutonymph, adult son) samples were pooled as *n* = 9 and *n* = 6, respectively. A *t* test was then performed and subjected to the Benjamini Hochberg correction at 5% FDR. All hierarchical clustering analyses were performed in Perseus using average Euclidian distance (300 clusters, maximum 10 iterations).

##### Functional Enrichment Analysis

We performed functional enrichment analysis on two sets of proteins: 1) *Varroa* proteins that were differentially expressed through development and 2) *Varroa* proteins that were differentially expressed between sexes. For all protein sets, we retrieved GO terms using Blast2GO (v4.0) with default parameters, first searching against all arthropods, then sequences with missing GO terms were searched again against the entire nonredundant protein collection. GO terms were exported after running the GO-Slim function. We then performed a gene score resampling analysis with ErmineJ v3.0.2 (37), using log-transformed q values (from the previous differential expression analysis) for protein score. We considered a GO term significantly enriched if the Benjamini Hochberg-corrected gene score resampling *p* value was less than 0.10.

##### Building the Varroa Protein Atlas

The web-based interactive *Varroa* protein atlas was built using the framework previously described for the honey bee protein atlas (29).

## RESULTS

### 

#### 

##### The New Varroa Gene Set has Dramatically Improved Accuracy Over the First Draft

**Fig. 2.**
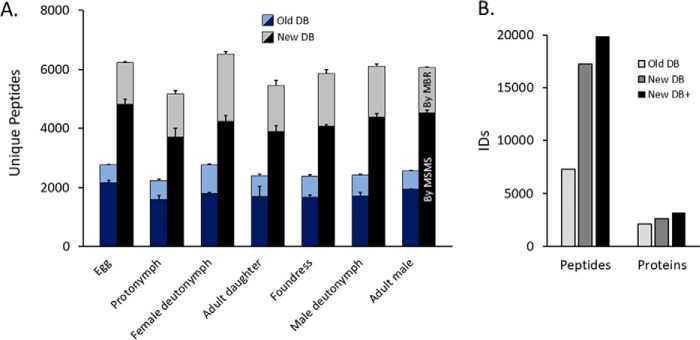
**Overall peptide and protein identifications.** MSMS data were searched against the initial draft *Varroa* gene annotation (old database) and the most recent updated annotation (new database). The data included biological triplicates of each developmental stage, and all protein databases also included NCBI *Varroa* sequences and all viruses known to infect honey bees and *Varroa*. (*A*) Light stacks represent peptide identifications via match between runs, and dark stacks represent identifications via MSMS matching. Error bars are standard deviation. (*B*) Cumulative identifications. New DB+ refers to the newest annotation plus all honey bee proteins and new fragments identified by proteogenomics.

**Fig. 3.**
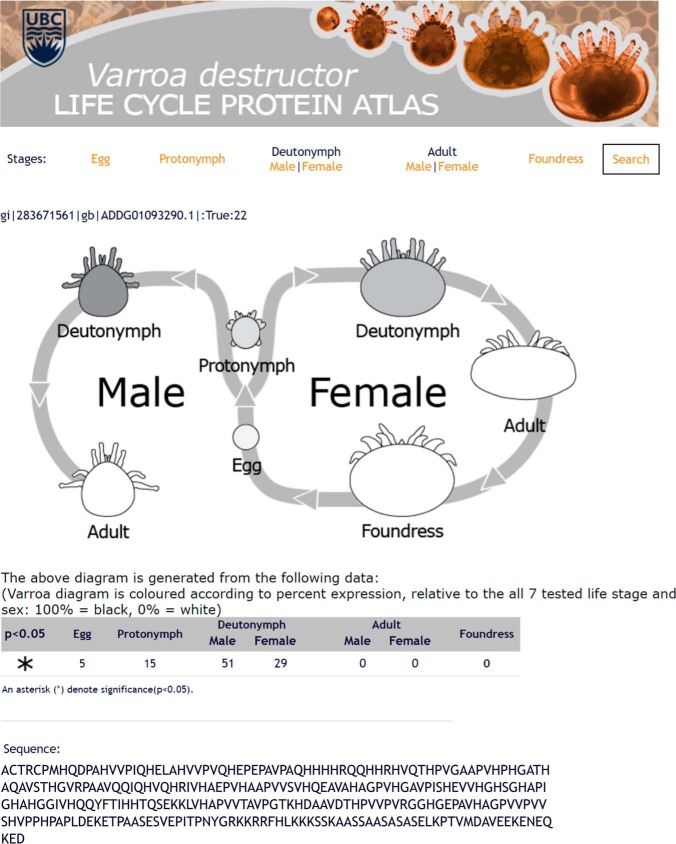
**Example page of the web-based V*arroa destructor* protein atlas.** The atlas was constructed using the framework described for the honey bee protein atlas (29). Shading of the cartoon mites indicates relative expression, and an asterisk indicates that this protein was significantly differentially expressed according to developmental stage. Website: http://foster.nce.ubc.ca/varroa/index.html.

Procuring an accurate protein database is critically important for proteomics applications. The first *Varroa* draft gene set was published in 2010 (1) along with the initial genome sequence (ADDG00000000.1); however, a new genome build was just released (ADDG00000000.2) with annotation refinement efforts underway.^1^ A new gene set will soon to be released, and we have made the new protein database provisionally available through ProteomeXchange (PXD006072). To test the accuracy of the new gene set compared with the first draft, we searched our complete *Varroa* proteomics data against both versions and found that greater than twofold more unique peptides were identified using the refined annotation (Fig. 2*A*). Overall, we identified nearly 20,000 unique peptides corresponding to 3,102 protein groups at 1% peptide and protein FDR (Fig. 2*B***)** representing the first global survey of *Varroa* protein expression. To maximize the utility of this information for researchers, we incorporated the quantified proteins into an interactive *Varroa* protein atlas (http://foster.nce.ubc.ca/varroa/index.html). The atlas features a searchable database of the quantified proteins as well as a visual and numerical display of their relative expression in different developmental stages (Fig. 3). All identified proteins, peptides and their corresponding information (accessions, scores, percent coverage, missed cleavages, *etc.*) are available in Supplemental Table 6.

##### Proteogenomics Identifies Unannotated Regions

**Fig. 4.**
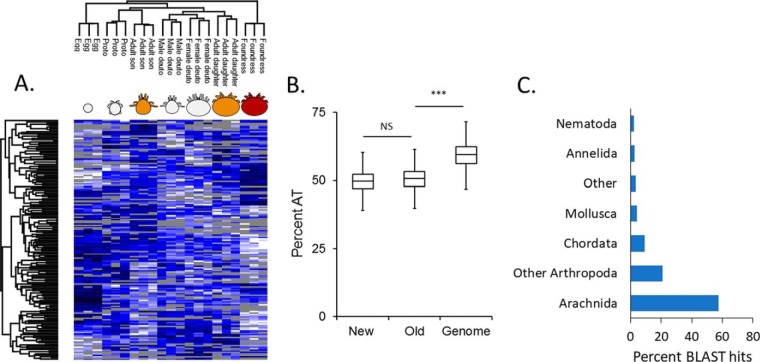
**A search against a six-frame genome translation database identifies new protein-coding fragments.** (*A*) New protein-coding fragments that are differentially regulated across development. Gray tiles represent missing data. Hierarchical clustering was performed in Perseus using average Euclidian distance (300 clusters, maximum 10 iterations). Statistics were performed using an ANOVA (Benjamini Hochberg-corrected FDR = 5%). (*B*) Comparison of the AT composition between newly identified sequences, previously known sequences (old) and the *in silico* fragmented genome. Statistics were performed using a one-way ANOVA (three levels) and a Tukey HSD post-hoc test. NS: not significant. ***: *p* < 0.0001. Boxes depict the interquartile range (IQR) and whiskers span 1.5*IQR. (*C*) BLAST sequence alignment summary of the new protein sequences for major (> 1% frequency) taxa.

Despite a dramatic improvement in accuracy over the initial draft annotation, the current annotation could likely be further improved through proteogenomics. We searched the MSMS data against a six-frame genome translation database and identified 519 protein-coding regions at 1% FDR (see Supplemental Table 1 for protein and peptide sequences) that were absent from the current annotation. 301 of these were supported by two or more peptides, representing high-confidence identifications. Furthermore, 169 of these protein groups were differentially expressed through development (Fig. 4*A*). This is in line with improvements we have discovered previously in *A. mellifera*, another nonmodel organism (34); however, since missed genes appear to be a common problem in genome annotation, we sought to investigate the root cause of failing to locate these sequences in the first place.

Gene prediction algorithms often use training gene sets from well-annotated species with similar genomic properties to help define genes in the newly sequenced target species (27). We hypothesized that one reason why an algorithm might fail to identify expressed sequences is if they occur in regions with significantly different adenine and thymine content or codon bias (indeed, this is precisely what happened during the *A. mellifera* annotation (38)), so we compared these properties between the newly identified protein coding regions and the previously known coding regions identified in the same six-frame translation search. We found that the newly identified regions had the same adenine and thymine content as the previously known regions, which were both significantly different from the genomic average (Fig. 4*B*). While this lends additional confidence that the new regions are expressed, it does not explain why they were missed. Furthermore, the amino acid composition and nucleotide positional codon bias (Supplemental Fig. 1) was the same between the new and known coding regions.

Since some algorithms rely on homology evidence to support annotations, one reason sequences may not be annotated is if they do not have known orthologs. We used Blast2GO to identify potential orthologs and found that nearly 72% (377) of the sequences had significant similarity (e-value cutoff: 1E-5) to at least one sequence in the nonredundant NCBI protein database (Fig. 4*C*). Of those, the majority (85%) matched to sequences from other members of phylum Arthropoda but Chordata, Nematoda, Mollusca, and Annelida were also present. Importantly, only nine sequences significantly matched well-annotated species (*Homo sapiens*, *Mus musculus*, *Drosophila melanogaster*, and *Caenorhabditis elegans*) and 148 (28%) had no significant sequence similarity to any species. Fifty-four of these sequences were supported by two or more peptides. In addition, five sequences were highly similar to known honey bee sequences, suggesting these are likely the result of DNA contamination within the *Varroa* sample used for DNA sequencing. This is not surprising since honey bee tissue is the mite's sole food source, so some contamination of this nature is expected. We removed these sequences since we include all honey bee proteins in our search database regardless in order to account for abundant honey bee proteins consumed by *Varroa*. All other fragments identified through proteogenomics were added to the protein database and utilized in subsequent analyses.

##### Vitellogenin, Carbohydrate Metabolism and Chitin Expression Underpin Developmental Transitions

**Fig. 5.**
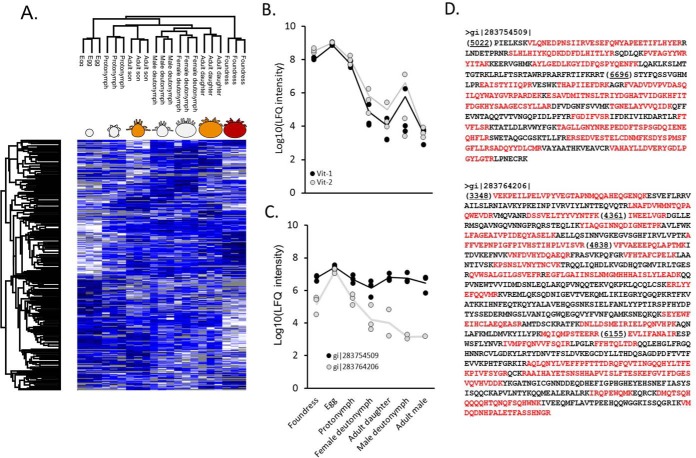
**Analysis of vitellogenin expression.** (*A*) Heatmap showing significantly differentially expressed proteins across developmental stages (ANOVA; Benjamini Hochberg-corrected FDR = 5%).Gray tiles indicate missing data. Proto and deuto refer to protonymph and deutonymph, respectively. (*B*) Vitellogenin (Vit)-1 and Vitellogenin-2 protein expression across developmental stages. (*C*) Expression of new protein fragments showing significant vitellogenin homology. (*D*) New vitellogenin protein fragment sequences. Observed peptides are red, the fasta header indicates the contig number, and bracketed numbers indicate the nucleotide start position of each fragment within the contig. Both protein sequences were coded on the reverse contig strand.

Of the 3,102 proteins identified, 1,433 were significantly differentially expressed across developmental stages (Fig. 5*A*; Table I). As a quality control method, we specifically analyzed vitellogenin (an evolutionarily conserved yolk protein) expression since this is one of the only proteins where the developmental patterns of expression are known (39). We expected to see high levels of vitellogenin-1 and vitellogenin-2 in the foundress and egg, with quantities decreasing approaching adulthood (39), and indeed, this is what was observed (Fig. 5*B*). Interestingly, some of the novel peptides identified in our proteogenomic effort mapped back to protein fragments with significant sequence similarity to vitellogenin, and upon closer inspection we found that some of these peptides are simply nonsynonymous single nucleotide sequence variants of this well-known gene. However, we also identified novel protein fragments with significant similarity to vitellogenin that did not physically overlap with the known vitellogenin genes (Fig. 5*C*). Like vitellogenin-1 and 2, the highest protein abundance for these novel sequences was in the egg. Furthermore, they group into two clusters of expressed fragments (one two-fragment cluster and one four-fragment cluster) closely linked on two different contigs, suggesting that the fragments form exons of two different genes (Fig. 5*D*) and clearly illustrate how mass spectrometry data can aid in gene predictions. Two of the significantly different proteins were viral polyproteins of deformed wing virus (DWV)^1^ and a *Varroa destructor* virus/DWV hybrid (Supplemental Fig. 2). The viruses displayed distinctly different expression profiles, with DWV appearing within the most abundant 1% of proteins out of all 2,626 proteins that were quantified—an extreme abundance for a pathogen-derived protein.

**Table I:** Summary of protein identifications

	Identified	Quantified	DEP	DEP* origins			
				*Varroa*	Virus	Bee	New
Development	3,102	2,626	1,433	1,148	2	114	169
Sex	3,000	2,260	101	86	0	1	14

* DEP, differentially expressed protein.

**Fig. 6.**
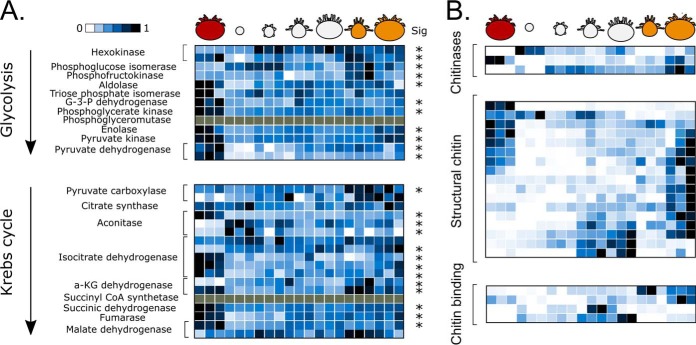
**Analysis of carbohydrate metabolism enzymes and cuticle proteins.** (*A*) Relative expression of enzymes involved in carbohydrate metabolism. Bracketed rows indicate isoforms of enzymes catalyzing the same reaction (based on shared enzyme codes and having the enzyme in question as the best BLAST hit). Gray tiles indicate the protein was not observed. Rows indicated with an asterisk are significantly differentially expressed across developmental stages (from Fig. 4*A*). G-3-P: glyceraldehyde 3-phosphate; a-KG: α-ketoglutarate. (*B*) Relative expression of proteins related to chitin formation. Only the significantly differentially expressed proteins are shown.

To gain a better understanding of the cellular processes underlying proteins that were differentially expressed through development, we performed an enrichment analysis by gene score resampling and found, not surprisingly, that lipid localization and lipid transport were among the most significantly enriched (Table II, Supplemental Table 2), driven largely by vitellogenin expression. Many processes involved in aerobic respiration were also significantly enriched, including GO terms linked to glycolysis (GO:0006090, GO:0006096) and the citric acid cycle (GO:006099, GO:0072350). To investigate these metabolic processes further, we analyzed how the abundances of core glycolysis and citric acid cycle enzymes varied with development (Fig. 6*A*). Most enzymes (16/20) were significantly differentially expressed and only two (phosphoglyceromutase and succinyl CoA synthetase) were not quantifiable. Several enzymes appear to have multiple isoforms, based on BLAST (Basic local alignment search tool) search results, some of which are not coexpressed (*e.g.* for hexokinase, α-ketoglutarate dehydrogenase, aconitase, isocitrate dehydrogenase, and malate dehydrogenase). Overall, the foundress mite has the highest levels of most enzymes, and when this is not the case, it is largely due to age-specific isoform expression. Relative expression levels for each protein can be found in Supplemental Table 3.

**Table II:** GO terms significantly enriched in developmental stages

Category	Name	ID	# Genes	Corrected *p* value*
Glycolysis & TCA	Aerobic respiration	GO:0009060	15	0.0777
	Carbohydrate metabolic process	GO:0005975	62	0.0556
	Dicarboxylic acid metabolic process	GO:0043648	10	0.0551
	Tricarboxylic acid cycle	GO:0006099	13	0.0509
	Glycolytic process	GO:0006096	10	0.0526
	Cellular respiration	GO:0045333	22	0.0531
	Monocarboxylic acid metabolic process	GO:0032787	36	0.0358
	Energy derivation by oxidation of organic compounds	GO:0015980	24	0.0343
	Tricarboxylic acid metabolic process	GO:0072350	14	0.0288
	Pyruvate metabolic process	GO:0006090	16	0.0179
	Generation of precursor metabolites & energy	GO:0006091	36	4.5E-10
	ADP metabolic process	GO:0046031	11	0.0544
	ATP metabolic process	GO:0046034	32	7.5E-11
	Ribonucleoside triphosphate metabolic process	GO:0009199	35	1.1E-10
	Purine nucleoside triphosphate metabolic process	GO:0009144	34	1.5E-10
	Nucleotide phosphorylation	GO:0046939	16	0.0056
	Nucleoside triphosphate metabolic process	GO:0009141	37	0.0064
	Purine nucleoside monophosphate metabolic process	GO:0009126	45	0.0149
	Purine nucleotide metabolic process	GO:0006163	49	0.0244
	Ribose phosphate metabolic process	GO:0019693	57	0.0261
	Purine-containing compound metabolic process	GO:0072521	51	0.0269
	Nucleoside monophosphate metabolic process	GO:0009123	48	0.0280
	Ribonucleoside monophosphate metabolic process	GO:0009161	47	0.0310
	Ribonucleotide metabolic process	GO:0009259	51	0.0348
	Nucleoside diphosphate phosphorylation	GO:0006165	12	0.0354
Amino acid metabolism	Aromatic amino acid family metabolic process	GO:0009072	7	0.0627
	Cellular amino acid metabolic process	GO:0006520	63	0.0512
Lipid movement	Lipid localization	GO:0010876	9	9.0E-11
	Lipid transport	GO:0006869	9	2.2E-10
Electron transport chain	Electron transport chain	GO:0022900	12	0.0694
	ATP biosynthetic process	GO:0006754	10	0.0973
Chemical homeostasis	Chemical homeostasis	GO:0048878	14	0.0504
	Cellular chemical homeostasis	GO:0055082	8	0.0720
Cation transport	Cation transmembrane transport	GO:0098655	35	0.0981
	Cation transport	GO:0006812	42	0.0728
Other	Protein deubiquitination	GO:0016579	8	0.0999
	Intra-Golgi vesicle-mediated transport	GO:0006891	5	0.0587

* Benjamini Hochberg-corrected enrichment *p* value.

Many proteins related to cuticle formation did not map to GO terms, despite having significant BLAST hits to chitin-like proteins. To analyze how cuticle formation may be developmentally regulated, we manually retrieved all proteins with significant BLAST hits to chitinases, structural chitin, and chitin-binding proteins. Indeed, we observed stark differences in the types of chitinases and structural chitin that are utilized (Fig. 6*B*). Young mites displayed a markedly different structural chitin profile than adult sons and daughters, which was different still compared with the armored foundress. Relative expression levels for each protein can be found in Supplemental Table 3.

##### Chromatin Remodeling and Positive Regulation of Transcription Underlie Sexual Differentiation

**Fig. 7.**
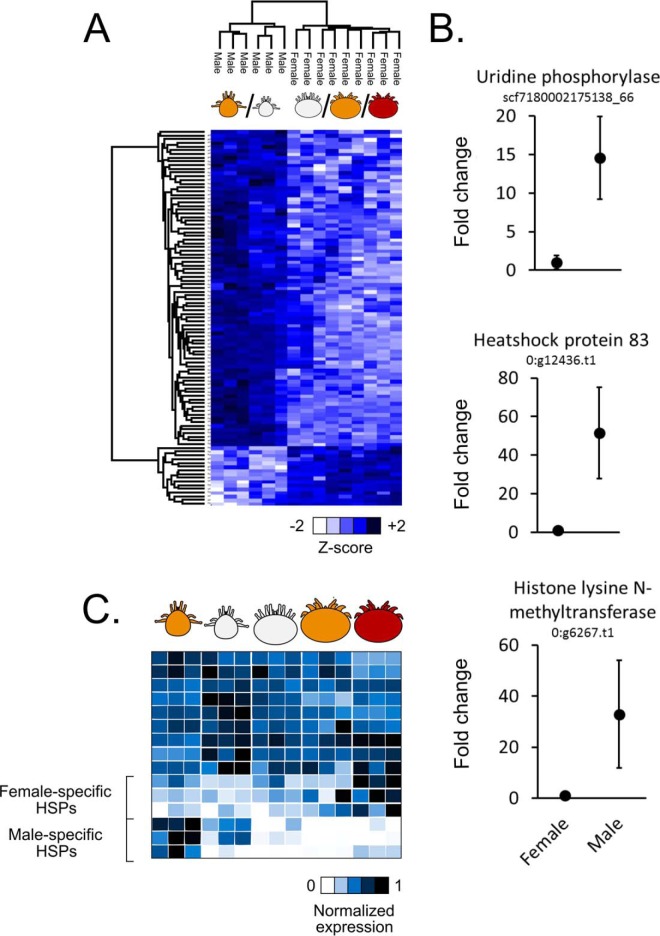
**Sexually regulated proteins in *Varroa*.** (*A*) Heatmap showing differentially expressed proteins in male (*n* = 6) and female (*n* = 9) mites (Benjamini Hochberg-corrected FDR = 5%). Hierarchical clustering was performed using average Euclidian distance (300 clusters, maximum 10 iterations). (*B*) The proteins with known functions among the top 10 differentially expressed. Fold change is normalized to the average expression in females. Error bars are standard deviation. (*C*) Relative expression of HSPs. Each row represents one HSP. Only significantly differentially expressed HSPs are shown.

*Varroa* follows the system of haplodiploid sex determination (*i.e.* females are diploid, males are haploid), but other than that, very little is known about the mechanisms that contribute to sexual differentiation. To investigate this, we compared the proteins expressed in female (*n* = 9) and male (*n* = 6) mites and found 101 starkly differentially regulated proteins, providing a starting point on which to further investigate possible differentiation mechanisms (Fig. 7*A*, Table I). A disproportionately large fraction (over 80%) of the differentially regulated proteins were up-regulated in the males. Investigating the 10 most significant proteins further, we found that only three had appreciable homology to sequences with known functions (Fig. 7*B*)—uridine phosphorylase, histone lysine N-methyltransferase, and heat-shock protein (HSP)83—while the others either had no significant sequence similarities or the significant matches have not been functionally annotated. Despite this, functional enrichment analysis revealed that GO terms relating to chromatin remodeling and positive regulation of transcription as well as various metabolic processes were significantly enriched (Table III, Supplemental Table 2). Intrigued by the prominent profile of HSP83, we further analyzed how the other HSPs are sexually regulated (Fig. 6*C*). We found that there is a core group of three HSPs that are specific to the foundress, and another group of three HSPs are male-specific.

**Table III:** GO terms significantly enriched in sexually regulated proteins

Description	Name	ID	# Genes	Corrected *p* value*
Chromatin remodeling	DNA packaging	GO:0006323	7	0.0970
	DNA conformation change	GO:0071103	11	0.0698
	Chromatin assembly or disassembly	GO:0006333	6	0.0833
Transcription	Positive regulation of gene expression	GO:0010628	6	0.0882
	Positive regulation of transcription, DNA-templated	GO:0045893	5	0.0945
Biosynthesis	Positive regulation of biosynthetic process	GO:0009891	6	0.0950
	Aromatic compound biosynthetic process	GO:0019438	70	0.0953
	Organophosphate biosynthetic process	GO:0090407	43	0.0967
	Nucleotide biosynthetic process	GO:0009165	37	0.0919
	Glutamine family amino acid biosynthetic process	GO:0009084	6	0.0750
	Organic acid biosynthetic process	GO:0016053	24	0.0798
Metabolism/catabolism	Cellular amino acid metabolic process	GO:0006520	64	0.0748
	Alcohol metabolic process	GO:0006066	5	0.0838
	Cellular nitrogen compound catabolic process	GO:0044270	13	0.0882
	Organic cyclic compound catabolic process	GO:1901361	16	0.0882
	Glycosyl compound metabolic process	GO:1901657	21	0.0882
	Nucleoside metabolic process	GO:0009116	20	0.0907
	Heterocycle catabolic process	GO:0046700	14	0.0937
	Nucleobase-containing compound catabolic process	GO:0034655	9	0.0937
Other	Peptidyl-amino acid modification	GO:0018193	34	4.4E-10
	Response to organic substance	GO:0010033	7	0.0682
	Response to oxygen-containing compound	GO:1901700	5	0.0762
	Protein folding	GO:0006457	48	0.0882

* Benjamini Hochberg-corrected enrichment *p* value.

##### Honey Bee Proteins Are Highly Abundant in Deutonymphs

*Varroa* mites feed on honey bee tissue, but how their diet might change during maturation is unknown. To examine how the honey bee proteins found within *Varroa* vary through development, we sorted out those proteins that were unequivocally honey-bee-specific (*i.e.* the majority protein ID (identification) within the proteinGroups.txt output only contained honey bee accessions) and found that, overall, the number and abundance of honey bee proteins was highest in the deutonymph stage (Supplemental Fig. 3*A*). We also observed a small group of honey bee proteins that were abundant mainly in the foundress, and we hypothesized that this could arise if the foundress consumes honey bee tissues other than the hemolymph. To investigate this further, we determined the overlap between the observed honey bee proteins and a representative honey bee hemolymph proteome (provided by Hu *et al.* (45); PXD004467). We found that ∼75% of honey bee proteins in *Varroa* originated from the hemolymph regardless of life stage (Supplemental Fig. 3*B*), so the origin of the remaining proteins remains unknown.

## DISCUSSION

The work presented here provides a foundation to begin to unravel the fundamentals of *Varroa* biology, including developmental transitions, sexual differentiation, diet, and host–virus interactions, as well as assisting with improving the genome annotation. Prior to this, there has been one published *Varroa* RNA-seq transcriptomics (24) and two proteomics studies, one of which—as far as we can tell—identified only virus and honey bee proteins and none from *Varroa* (15), and the other only analyzed foundresses (26). With almost 20,000 unique peptides identified, our study represents the deepest *Varroa* proteome to date. Overall, we identified 3,102 proteins, 2,626 of which were quantified by LFQ and incorporated into an interactive web-based *Varroa* proteome to serve as a community resource.

Genome sequencing is becoming relatively easy, but accurately annotating the genome is an arduous and imperfect process. The most common model organisms (*e.g. M. musculus*, *D. melanogaster*, *C. elegans*, *etc.*) have benefitted from decades of genetic research that has refined their genome annotations over time, resulting in highly reliable and accurate gene sets on which most tools for analyzing global gene and protein expression rely. Our data clearly show that the new *Varroa* gene annotation is far better than the provisional draft (Fig. 2), but our proteogenomics initiative, which identified 1,464 unique unannotated peptides, suggests that there is still room for improvement. While some of these novel peptides simply harbor nonsynonymous sequence polymorphisms, that itself is worth reporting, and this information can be used to augment the protein databases used for mass spectrometry searches (46). Other peptides, however, clearly corresponded to exons of unannotated genes (Figs. 5*C* and 5*D*) that showed significant homology to vitellogenin. This observation, along with finding nothing unusual about the sequence properties of the newly identified coding regions (Fig. 4*B*, Supplemental Fig. 1) led us to question why they were not already annotated.

The annotation process is not only influenced by the genome itself (chemical and physical properties, completeness, *etc.*) but also by the quality of guiding transcript assemblies and a number of human-determined parameters (*e.g.* the annotation software employed, hard or soft repeat masking, splice site awareness, *etc.*), and availability of prior gene models (47, 48). Furthermore, some parameters may need to be altered on a species-by-species basis, but there is no inherent pathway for finding the optimal settings. Proteomics and RNA-seq data could serve as tools to not only confirm expression of predicted genes but also to help define these parameters in the first place since the resulting protein and gene IDs are sensitive to database accuracy. The data we present here are all publicly available (PXD006072), and we urge future iterations of annotation refinement to take full advantage of this peptide evidence when developing new *Varroa* gene models.

In mass-spectrometry-based proteomics, it is important that the protein database reflects the proteins that could be present in the sample. Since *Varroa* feeds on honey bee tissues and others have detected honey bee proteins in *Varroa* (15), we included honey bee proteins in the search database and found that 167 of them were significantly differentially abundant (Supplemental Fig. 3). The eggs were largely lacking in honey bee proteins, which is in keeping with the developing embryos not yet being able to feed on wounded honey bee pupae. The presence of some honey bee proteins in the egg suggests these are contamination; however, the deutonymph stage of both sexes, which are actively feeding on hemolymph, showed the highest abundance of honey bee proteins. This suggests that the deutonymphs require large amounts of food, possibly to support energetically expensive developmental processes such as metamorphosis.

Our analysis of developmentally regulated proteins revealed some intriguing trends regarding the energetic demands throughout development (Fig. 6*A*). The foundress had consistently high abundances of enzymes that participate in glycolysis and the citric acid cycle, which may be required to meet the energetic demands of producing and laying eggs. We speculate that many of the differences in metabolic processes are also driven by the unique energetic requirements of metamorphosis, when energetically expensive morphological rearrangements must occur while the mite does not eat.

During maturation, protonymph and deutonymph mites transition from having a soft, translucent cuticle to acquiring a harder and more durable exoskeleton. The phoretic and foundress mites have rigid armor to protect against injury by grooming honey bees and other environmental hazards. To investigate the possible mechanisms behind these transitions, we compared the expression profiles of significantly differentially expressed proteins that are related to cuticle development (chitin structural protein, chitinases, and chitin-binding proteins; Fig. 6*B*). The egg contains large amounts of one chitinase and one chitin structural protein, which could be related to the breakdown of the egg case or the developing mite larva as it becomes a protonymph. Deutonymphs display a specific profile of highly abundant structural proteins and chitin-binding proteins, and from this point on, there is a clear separation between male and female expression profiles. The male mite appears not to invest energy in forming a tough exoskeleton like the female does, which is consistent with the lack of environmental exposure during the male life cycle.

In our analysis of sexually regulated proteins, we found that chromatin remodeling and transcription activation were significantly enriched processes. Chromatin remodeling could be required to decondense chromosomal regions that are highly expressed in males or females and *vice versa*. Indeed, histone lysine N-methyltransferase was one of the most significant differentially expressed proteins, with ∼30-fold higher levels in males compared with females (Fig. 7*B*), and peptidyl-amino acid modification was the most significantly enriched biological process (Table III). This kind of on–off regulation could thus be very important for sex determination. We also found that HSP83, which is critically important for spermatogenesis in *Drosophila* (49), displayed the greatest fold change (∼50-fold) out of those with known functions. Broadening our analysis to all identified HSPs, we found that there is a core group of HSPs that are specific to the foundress and another group that is specific to males (Fig. 7*C*), suggesting that these HSPs are involved in regulating the transcription of sex-specific genes.

The work we present here represents a first-of-its-kind, high-resolution analysis of the *Varroa* proteome. With some 1,433 proteins that are differentially expressed, these data provide a first glimpse into the changes that take place during *Varroa* development. In addition, 101 strongly sexually regulated proteins provide clues for discovering the mechanisms behind sex determination and general dimorphism. We hope that the interactive web tool will maximize the utility of this information for the research community and will help generate further hypotheses for future experiments on this major honey bee pest.

## DATA AVAILABILITY

Raw mass spectrometry data can be downloaded from the PRIDE Archive (www.ebi.ac.uk/pride/archive/), accession PXD006072. Annotated spectra are available through MS-viewer (http://msviewer.ucsf.edu/prospector/cgi-bin/msform.cgi?form=msviewer) with search keys wuh30b9smr and msmx6z444s.

## Supplementary Material

Supplemental Data

## Footnotes

^1^ The abbreviations used are:

DWV: deformed wing virus
FDR: false discovery rate
Jay Evans: personal communication (2017)
BMLV: Bee macula-like virus
VDV: Varroa destructor virus
MBR: match between runs
AT: adenine and thymine
DB: database
DEP: differentially expressed protein
GSR: gene score resampling
ID: identification
HSD: honestly significant difference
BLAST: basic local alignment search tool.
