# Supplementary material for: A Varroa destructor protein atlas reveals molecular underpinnings of developmental transitions and sexual differentiation
Source: Mol Cell Proteomics. 2017 Sep 22;16(12):2125–37. doi: 10.1074/mcp.RA117.000104 (PMC5724176; doi:10.1074/mcp.RA117.000104)
Supplement: Supplemental Data [file supp_16_12_2125__index.html]

A Varroa destructor protein atlas reveals molecular underpinnings of developmental transitions and sexual differentiation — A Varroa destructor protein atlas — A Varroa destructor protein atlas reveals molecular underpinnings of developmental transitions and sexual differentiation — A Varroa destructor protein atlas — Supplemental Data 

# A *Varroa destructor* protein atlas reveals molecular underpinnings of developmental transitions and sexual differentiation

## Supplemental Data

- Supplementary Figures S1-S3 - Proteogenomics analysis of sequence properties (S1), virus protein expression across developmental stages (S2) and honey bee protein abundance across developmental stages (S3)
- Perl scripts used for proteogenomics analysis - These scripts were used to analyze the sequence properties (amino acid frequencies, codon bias, nucleotide bias) between our newly identified sequences and known Varroa sequences
- Novel protein and peptide sequences identified by the proteogenomics search - Novel protein sequences identified via the 6-frame translation search and their corresponding peptide sequences (both at 1% FDR)
- Functional enrichment analysis results - Ermine J was used to perform functional enrichment on proteins significantly differentially expressed between developmental stages and sexes
- TCA, Glycolysis, Cuticle and HSP expression - TCA, Glycolysis, Cuticle and HSP normalized expression values based on LFQ data.
- Bee protein abundance and GO terms found within deutonymphs and foundresses - Bee protein abundance is based on LFQ data and GO terms were retrieved with BLAST2GO
- Proteogenomics protein expression, number of unique peptides, and protein coverage - The MaxQuant proteinGroups.txt file resulting from the 6-frame translation proteogenomics search
- Protein expression, number of unique peptides, protein coverage and LFQ intensities - The MaxQuant proteinGroups.txt file resulting from searching against the canonical (including bee, virus, mite and novel sequences identified by proteogenomics) protein database. Differential expression analysis used 'normalized LFQ intensities'
