# Supplementary material for: A Varroa destructor protein atlas reveals molecular underpinnings of developmental transitions and sexual differentiation
Source: Mol Cell Proteomics. 2017 Sep 22;16(12):2125–37. doi: 10.1074/mcp.RA117.000104 (PMC5724176; doi:10.1074/mcp.RA117.000104)
Supplement: Supplemental Data [file supp_RA117.000104_4674_1_supp_4433_22253z.docx]

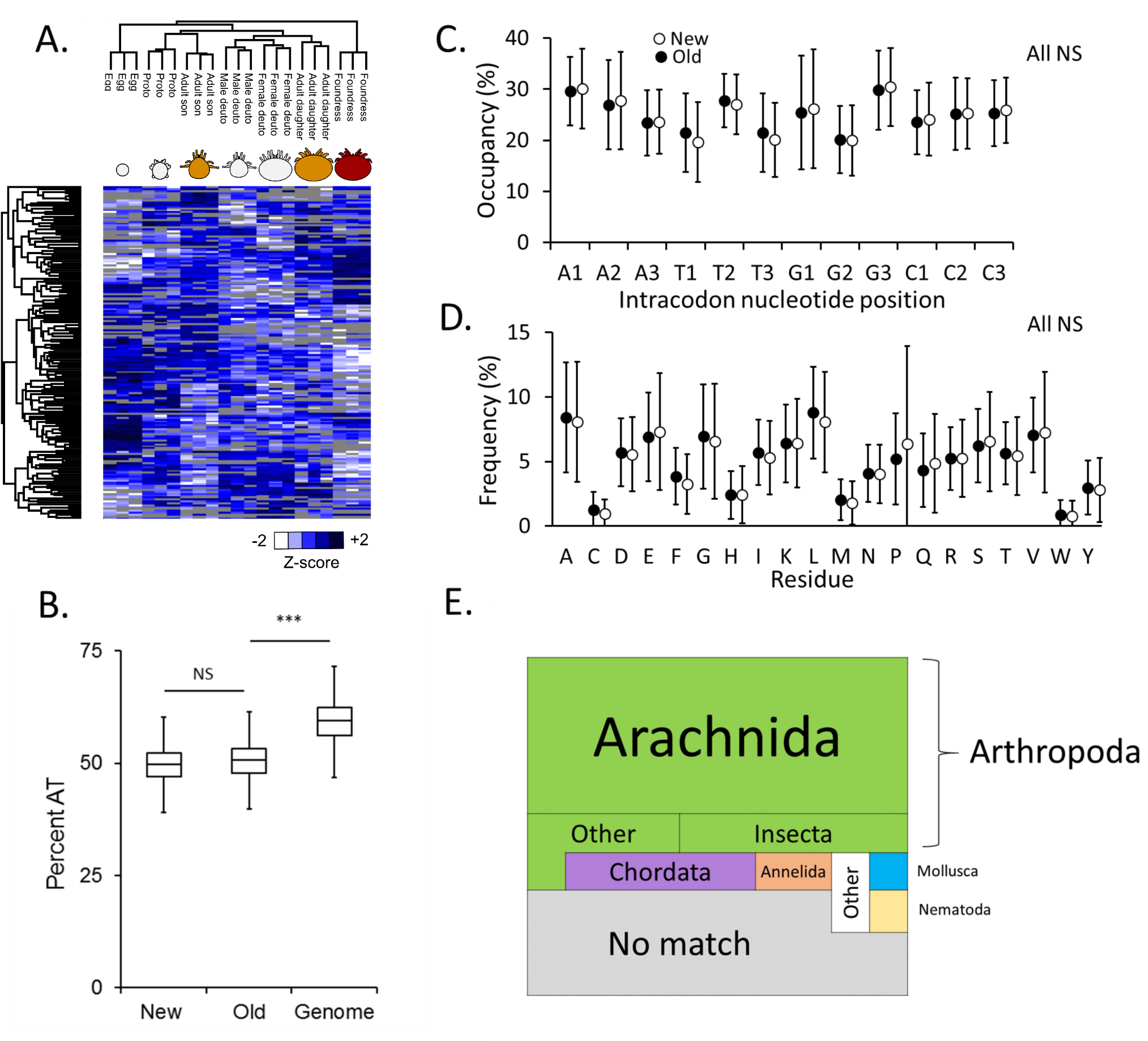


Supplementary Figure 1. *Comparison of nucleotide and amino acid residue frequencies between new and old* Varroa *sequences.* There are no significant differences in mean amino acid composition or intracodon nucleotide position of new protein coding regions compared to old (one-way ANOVA). Error bars represent standard deviation.


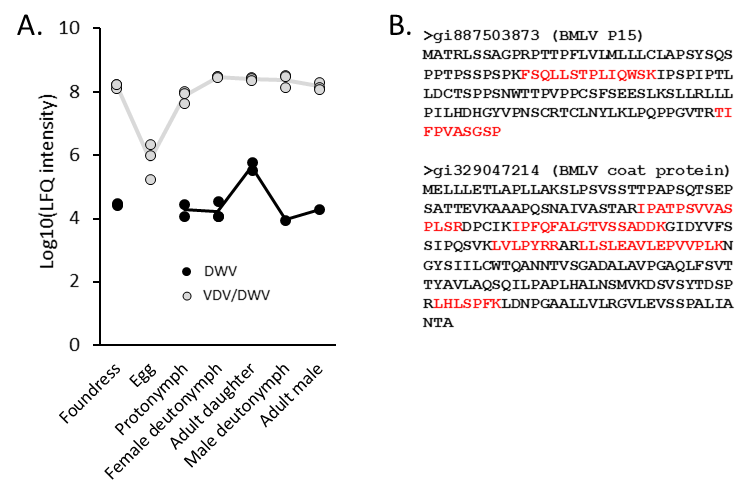


Supplementary Figure 2. *Survey of viral proteins*. A) Deformed wing virus (DWV) and *Varroa destructor* virus (VDV)/DWV (a strain distinct from the aforementioned DWV) were significantly differentially expressed across developmental stages (ANOVA; Benjamini Hochberg-corrected 5% FDR). B) Bee macula-like virus (BMLV) proteins (1% protein and peptide FDR in MaxQuant). Red regions represent observed peptides.

**
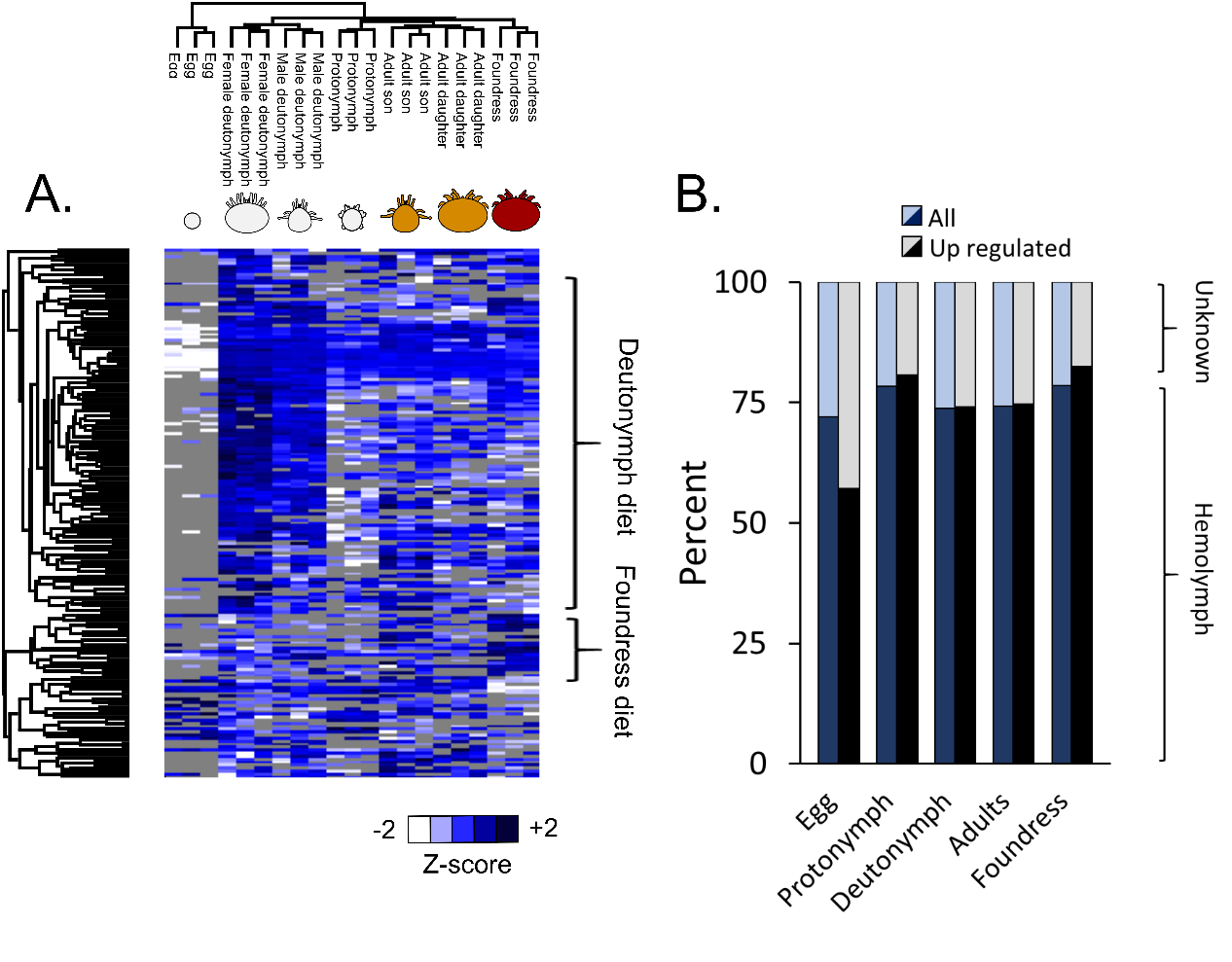
**

**Supplementary Figure 3**. *Honey bee proteins which were differentially abundant throughout* Varroa *development*. A) Grey tiles are missing data. Differential abundance is based on Benjamini Hochberg-corrected 5% FDR. Hierarchical clustering was performed using average Euclidian distance (300 clusters, maximum 10 iterations). “Deutonymph diet” and “foundress diet” indicate distinguishable clusters of proteins which are highly abundant in those developmental stages. B) Fraction of bee proteins originating from the pupa hemolymph (dark bars) and unknown sources (light bars).
